# Supplementary material for: Are carbon emissions trading and green financial instruments synergistic? -Comprehensive quantitative research based on content analysis
Source: PLoS One. 2024 Mar 7;19(3):e0298601. doi: 10.1371/journal.pone.0298601 (PMC10919601; doi:10.1371/journal.pone.0298601)
Supplement: S1 Data — (ZIP) [file pone.0298601.s001.zip › Data/地级市Z_F工作报告.zip!/╔╜╬≈/╜·│╟╩╨/└┤╫╘╣1⁄2╓┌║┼í╛┬φ┐╦╩2╛▌═°í┐_2009.docx]

过去一年的主要工作

过去的一年是我市经济社会发展迎难而上、奋勇拼搏，取得显著成绩的一年，也是市人大常委会工作不断完善制度、取得明显实效的一年。在市委的领导下，常委会认真贯彻落实党的十七大和十七届三中全会精神，深入学习实践科学发展观，切实履行宪法和法律赋予的各项职权，圆满完成了市五届人大五次会议确定的各项工作目标。

一、围绕中心促发展，提升监督工作水平

一年来，常委会根据监督法的要求，以发展为目标，寓支持于监督，按照围绕中心、突出重点、注重实效、提升水平的工作思路，听取和审议“一府两院”专项工作报告8个，审议落实情况书面报告7个，督促和支持政府解决转型发展、安全发展、和谐发展中的突出问题，有力地促进了全市经济社会稳定协调较快发展。

（一）围绕转型发展实施监督。我市因煤而兴，为煤而困，调整经济结构，实现转型发展是我市的一个重大课题。为此，常委会在监督转型发展上，坚持狠抓“四个重点”：一是把“十一五”规划的中期评估作为监督重点，认真组织了“十一五”规划实施情况的专题调研，听取和审议了市政府的专项报告，明确要求政府调整“十一五”后期指标体系，实现传统产业抓升级，新型产业上规模，结构调整抓三产，企业发展重民营，推动我市经济战略转型加快步伐。二是把节能减排工作作为监督重点，专题听取和审议了市政府关于煤化工基地建设情况的报告。通过督促落实审议意见，推动市政府加大煤化工园区的规划及配套建设力度，加快了煤化工业废水零排放技改工作的推进速度，有力地促进了我市煤化工产业的上等升级。三是把前半年经济工作和农业服务体系情况作为监督重点，专题听取和审议了市政府关于2008年1—7月份经济运行情况、建立健全农业科技和服务体系情况两个专题工作报告。市政府按照审议意见的要求，召开了全市加快服务业发展大会和全市民营经济工作会议，确定了全市发展服务业的“1850”工程建设目标，加快了农业服务八大体系建设，并拿出1亿元财政专项资金，用于支持工业技术进步，积极推广应用节能减排适用技术，扎实推进了转型发展进程。四是把城市发展战略的总体规划作为监督重点，听取和审议了市政府城市总体规划报告，要求规划要具有前瞻性，彰显科学性，体现权威性，维护严肃性，使城市总体空间布局实现了从“一城两区”到“六区联动”的历史性跨越，目前，市区“两抓四射六横七纵”的城市路网工程正在积极推进，为把我市建设成为现代化宜居城市奠定了坚实基础。

（二）围绕安全发展实施监督。安全是生存的基础、发展的前提。我们要加快发展，必须首先抓好安全发展。为此，常委会围绕安全发展，坚持“两抓两保”：一是抓执法检查，保障生产安全。常委会听取和审议了执法检查组关于检查水污染防治法实施情况的报告，并分别审议了市政府落实市人大常委会执法检查组关于检查安全生产法、水法实施情况报告整改意见的两个书面报告，监督政府进一步完善了安全生产体系，落实安全责任，强化隐患治理，使全市安全生产依法有序，保持了良好态势。二是抓“治水”监督，保障生态安全。首先，完成了丹河条例的修订工作。常委会针对近年来我市丹河沿岸工矿企业发展迅猛、承载能力超限的严峻现实，反复呼吁，积极创造条件，努力做好各项基础工作，促使《山西省丹河流域水污染防治条例》的修订工作列入了省人大常委会2008年立法议程。常委会多次配合省人大组织实地勘查，并召开各种征求意见会、专家论证会数十次，经过坚持不懈的努力，终于使这一部富有鲜明晋城特色的地方性法规于2008年11月28日经省人大常委会第二审通过，并于2009年1月1日颁布施行，夯实了治理丹河、保护丹河的法制基础。其次，连出重拳，保障合理安全用水。常委会认真监督落实第十九次会议作出的《关于市区水资源保护和合理开发利用的决议》，到2008底，封闭市区生活用水不达标的浅中层水源井目标已基本实现，为关闭市区自备井迈出了关键一步。在此基础上，常委会又组织了市区河流补充水源专题调查，提出了外引水源，内治河道的补充水源构想，为优化我市水环境和促进水资源的科学开发利用起到了积极的推动作用。坚持15年的“晋城环保行”活动，2008年以“净空气、美环境、迎奥运”为主题,以检查采访的监督措施，促进了治污进程，使全市集中式饮用水源地水质达标率达到了100%。第三，专题视察了白马寺山森林公园地质灾害情况。针对近年来白马寺山森林公园发生的地质灾害情况，组织部分市人大代表进行了专题视察，提出了“对白马寺山地质状况进行科学勘探和评估、科学开发建设白马寺山森林公园、责成有关企业承担灾害治理责任、下决心逐步关闭城市规划区范围内煤矿”等五条建议，为白马寺山森林公园的灾害治理、生态保护和科学开发提供了重要的客观依据。

（三）围绕和谐发展实施监督。常委会以促进社会和谐为己任，把监督的着力点放在“四促一保”上：一是促进社会保障。首先，城市保住房。常委会先后听取和审议了市政府落实常委会关于建立健全农村社会保障机制情况报告审议意见和住房保障体系建设情况两个专项工作报告。在常委会的有效监督下，我市住房保障政策体系逐步完善，保障性住房建设取得突破性进展，全市全面启动了经济适用房、廉租住房建设和城市居民棚户区改造工作，从源头上满足了困难家庭特别是低保户等弱势群体“有钱租房、租得上房”的基本需求。其次，农村保医疗。常委会认真督促落实市五届人大五次会议关于新型农村合作医疗专题审议意见，促进政府加大了政策宣传、体制完善和安全监管，新型农村合作医疗在扩大受益范围、加强资金监管、提高报销比例、降低医疗价格、扩大门诊补偿、改善基层医疗设施和提升乡村医疗服务水平等方面，走在全省的前列，受到了广大农民群众的欢迎。二是促进教育、文化发展。常委会在过去几年连续监督教育工作的基础上，继续加大检查和落实力度，审议了市政府落实市人大常委会关于“十一五”市区中小学教育发展规划及实施意见的书面报告。督促市政府加大中小学校舍建设，收到明显效果。到目前为止，市直学校建设项目进展顺利，校际教学资源均衡提升，教师队伍充实加强，教学质量也有了进一步的提高。常委会听取和审议了市政府关于农村文化建设情况的报告，在常委会的积极推动下，启动了农村文化基础设施建设，推动了各类文化资源的整合，增加了文化产品和服务的供给总量。三是促进政务环境改善。常委会听取和审议了市政府关于落实执法检查组关于行政许可法实施情况报告整改意见的书面报告，支持政府再次取消了17项行政审批项目，建立了撤销许可的补偿机制，加大了对行政许可行为的监督检查力度，进一步改善了政务环境。四是促进民调工作。常委会听取和审议了市政府关于人民调解工作情况的报告，促进了民调经费的落实、机构队伍建设和办公条件的改善，基层民调工作出现了新的局面，为建设“和谐晋城”起到了积极的推动作用。五是保障惠民工程落实到位。常委会对市政府为民办的十六件实事进行了认真的检查监督，联合市纪委效能监察部门和市政府督查部门，以一月一检查、两月一公示的跟踪监督措施，督促解决有关问题，促进了十六件惠及民生的实事项目圆满完成。

在认真实施工作监督的同时，常委会还在全省率先出台了《晋城市人大常委会规范性文件备案审查暂行办法》，对市人民政府和县（市、区）人大常委会向市人大常委会申报的规范性文件初步开展了备案审查工作，使法律监督工作迈开了新的步伐，为保障国家法制统一发挥了积极作用。

二、抓住关键保重点，依法决定重大事项

常委会认真行使重大事项决定权，紧紧围绕全市工作大局，抓住大事，认真审议，依法作出决议4项、决定3项，为保障和促进全市经济社会协调发展，发挥了国家权力机关的职能作用。

（一）抓住关键，加强计划预算审查。计划、预算的批准和执行情况以及财政决算情况，事关国计民生，既是常委会监督的重点，也是常委会依法决定重大事项的关键内容。为此，常委会紧紧抓住两个关键环节：一是初审体现民主性。为促进科学合理安排全市年度预算，市人大财经委提前介入计划预算编制工作，组织常委会组成人员、财经委委员和财经咨询组成员听取经济工作部门和项目单位的汇报，广泛征集社会各方面意见和建议，在充分集中民智的基础上，提出了针对性的初审意见，为人代会决定全市经济和社会发展的“大盘子”提供客观依据。二是决议维护严肃性。在去年作出的关于2007年度市本级财政决算的决议中，常委会明确要求市政府要努力实现财源结构多元化，规范部门预算管理、强化项目库建设，规范政府投资项目管理，努力提高资金使用效益；对审计报告中反映的问题，要求从体制和机制上分析原因，提出改进措施。按照《决议》要求，2008年审计查出问题的整改工作取得了明显成效，现已收缴整改资金803.27万元，征收应征的财政收入78598.47万元，依法维护了人代会批准预算和常委会决议的严肃性，推动了市政府依法理财、科学理财、为民理财的进程。

（二）关注重点，保障项目落实。充分利用银行贷款支持地方建设，是近年来我市借鉴兄弟省市经验的有益尝试。2006年12月市人大常委会第三次会议作出决议，同意市人民政府利用国家开发银行贷款进行城市公用基础设施建设，其中，市东南出入口生态修复工程项目已荣获2008年“中国人居环境范例奖”。根据市五届人大五次会议确定的重点工程和市政府提出的利用银行贷款的报告，2009年2月常委会第24次会议又作出了《关于批准市政府利用国家农业银行贷款建设晋城学院的决议》，要求市政府积极稳妥地利用好银行贷款，严格按照常委会批准的用途使用，项目完成后要进行严格审计，并向市人大常委会作出专题报告。

常委会坚持党管干部和人大依法任免国家机关工作人员的统一，认真行使人事任免权，决定任免和任免国家机关工作人员37人次，依法圆满完成了人事任免任务，并进一步强化任期监督，促进了国家机关工作人员的勤政廉政建设。

三、创新方式求实效，充分发挥代表作用

一年来，常委会不断创新代表工作的方式方法，在组织代表活动上不断拓展新途径，进一步发挥了代表作用，使全市人大工作基础更加坚实。

（一）指导代表小组活动有新举措。常委会在全国人大北戴河培训中心举办了市人大代表活动小组组长培训班，近百名代表活动小组组长及部分市人大代表参加了培训。常委会还指导代表活动小组建阵地、立制度，结合各地实际，联系民情民意开展活动，促进解决实际问题，得到了选区选民的充分认可。常委会组织开展了争创先进代表小组和优秀人大代表活动，在新闻媒体上对优秀代表事迹进行了巡回展示，并将在本次会议期间对选出的6个先进代表小组和38名优秀人大代表进行表彰，进一步激发了代表履职的积极性。

（二）组织代表活动有新特色。常委会坚持完善和落实组成人员联系代表、代表联系选民、代表活动情况通报、代表向选民述职等制度，密切代表和人民群众的联系，接受选民监督。常委会印发了《关于人大代表要认真学法、模范守法的通知》，规范了人大代表带头学法守法、依法履职的行为；组织了代表跨选区交叉视察活动，拓展了代表的知情知政视野；以《代表论坛》为载体组织代表开展了大规模的专题调研活动，并写出了195篇调研报告。还在评出的优秀论文中，与电视台联合举办了“新农村建设”、“大力发展循环经济”及“人大代表的‘位’与‘为’”三个现场访谈形式的专题节目，展示了代表调研成果，并举行了总结表彰大会。一年来常委会邀请34名代表列席了常委会会议，邀请100余名代表参加了常委会及各专工委组织的专题调研和执法检查活动，充分发挥了代表作用。

（三）办理代表建议有新成效。常委会坚持“以提高办理质量为重点，以代表满意为标准”，不断完善督办机制，强化督办措施，坚持实行了副市长领办代表重点建议、常委会副主任和对口工委督办代表建议制度，分别听取和审议了市政府和市中级法院关于市五届人大五次会议代表建议办理情况的报告。常委会实行了重点建议月报制度，市电视台《代表之声》制作播出了24期办理代表建议节目，在市广电中心新闻发布厅召开了代表建议办理情况通报会，将276件代表建议办理检查情况进行了集中公布，对8件办理成效不明显、代表不满意的建议进行了通报，使办理代表建议的“面商率、解决率、满意率”有了新的提高。

为加强对基层人大工作的指导，常委会本着“加强联系、加强指导、加强服务”的原则，在沁水县召开了全市乡镇人大工作座谈会。会议总结交流了近年来我市乡镇人大工作经验，并就新形势下如何进一步改进和加强乡镇人大工作进行了深入研讨，对全面提升全市基层人大工作整体水平发挥了积极作用。

四、“四位一体”树形象，全面推进自身建设

一年来，常委会按照政治坚定、业务精通、务实高效、作风过硬、团结协作、勤政廉洁的要求，紧紧围绕“服务、规范、创新、活力”这一主题，坚持“四位一体”树形象，全面推进了常委会及人大机关工作的规范化、制度化建设。

（一）学习教育重制度，保实效。坚持举办常委会法制讲座，认真组织每周一下午的机关集中学习制度，一年来，先后就十七届三中全会精神、文化建设、社会建设等举办了5期法制讲座，邀请全国人大专家举办了《中华人民共和国水污染防治法》大型法制讲座，组织机关集中学习28次，通过建立和完善学习考核制度、通报制度和相互交流学习体会制度，保证了学习效果。坚持常委会组成人员履职考核制度，增强了组成人员履职的责任意识，履职能力有了新的提高。目前，人大机关正在按照市委的统一部署，积极开展深入学习实践科学发展观活动。

（二）宣传工作扩阵地，出成果。年初，常委会召开了全市人大宣传工作会议，全面部署了人大宣传工作目标任务。根据监督法要求，坚持了会议及重要活动向社会公布制度，并进一步扩阵地、拓渠道，创办了太行日报《人大专刊》，及时向社会公布人大监督工作情况，接受社会各界的监督。常委会创办的《晋城人大》期刊，成为了2008年全国人大图书馆特色图书馆藏刊物。同时，晋城人大网站和电视台《代表之声》栏目不断创新，一批宣传人大制度和人大工作的重要文稿先后在《人民代表报》、《中国人大》、《山西人大》等报刊上发表，并被河北人大网、人民代表网等网站转载，起到了宣传人大、促进工作的目的。

（三）信访工作保重点，求实效。常委会继续坚持和完善信访联席会议制度，对一般案件及时转办，重点涉法案件采取联席会议交办、一督到底的办法，下大力抓好督办，全年共交办涉法涉诉信访件7件，现已办结5件。一年来，常委会受理人民群众来信 35 件，接待来访987人次，基本做到“件件有着落、事事有回音”。常委会还积极探索信访工作的规范化和制度化建设，年终召开全市人大信访工作座谈会，就信访工作的体制、机制进行了有益的探索和研究。

（四）机关管理抓规范，明职责。常委会全面修订了机关管理制度，完善了各工作机构、办事机构职责和工作人员岗位责任，进一步强化和提升了机关服务“三会”的效能。常委会党组高度重视机关党建工作，加强了机关思想、组织建设，深入开展创建和谐机关活动，在抗震救灾、向红十字会献爱心等活动中，人大机关先后四次捐款19360元，缴纳特殊党费59000元，机关文明和谐建设呈现出蓬勃向上的新面貌。

同时，常委会十分重视对外友好交往和工作交流，先后配合省人大常委会在我市举办了全省代表工作座谈会，应邀参加了全国23城（市、区）人大常委会联席会议和24城市人大工作研讨会议，全年接待兄弟省市人大来访客人85批、近2000人次，赢得了良好社会声誉。

在取得成绩的同时，我们也清醒地看到，常委会的工作中还存在许多不完善、不全面的地方。主要是：在增强工作的实效上，常委会审议意见跟踪监督的力度还需进一步加大；在提高专项报告的审议质量上，审议的方式方法还需进一步创新；在加强常委会的自身建设上，求真务实的作风、调查研究的基本功还需进一步提高；在提高机关效能上，各项制度还需进一步落实。对于这些差距和不足，在学习实践科学发展观活动中一定要认真研究，努力改进。

各位代表，过去一年常委会工作所取得的成绩，是市委正确领导的结果；是全体市人大代表、常委会组成人员以及机关工作人员辛勤工作的结果；是市人民政府、市中级人民法院、市人民检察院及有关方面密切配合、广大人民群众大力支持的结果。在此，我受市五届人大常委会的委托，向大家表示崇高的敬意和衷心的感谢！

面对新的形势、新的任务和新的要求，实践使我们深深体会到，要做好新时期的人大工作，必须切实把握好以下几个方面：

（一）必须坚持人大工作正确的政治方向，把党的领导贯穿人大工作始终。坚持正确的政治方向是做好人大工作的根本。人大工作坚持正确的政治方向，最根本的是坚持党的领导、人民当家作主、依法治国的有机统一，核心是坚持党的领导。实践证明，只有自觉接受党的领导和主动争取党委支持，人大工作才能有力度、有影响、有实效。这些年来，常委会始终坚持认真贯彻执行市委的重大决策，围绕市委中心工作履行职责；坚持重大问题向市委请示报告；坚持把党的领导落实到人大依法行使职权上，努力把市委的主张通过法定程序变成全市人民的共同意志，把党管干部原则和人大依法任免国家机关工作人员有机结合起来，确保了常委会的工作沿着正确的轨道健康运行。

（二）必须坚持围绕中心履行职责，把促进发展贯穿人大工作始终。发展是执政兴国的第一要务，也是我市解决一切问题的前提和基础。常委会把促进本行政区域经济和社会发展作为全部工作的中心任务，在确定监督工作计划和安排工作要点时都事先征求“一府两院”的意见，力求在工作目标的制定上同向合拍；在审议专项报告、决定重大事项、开展执法检查时，把中心工作作为重点，力求在工作过程中同心合力，紧紧围绕中心不动摇，促进发展求实效，从而为促进全市经济社会又好又快发展发挥了权力机关的应有作用。

（三）必须坚持以人为本、执政为民，把民本理念贯穿人大工作始终。人大常委会来自于人民，必须对人民负责，受人民监督，要始终把群众的冷暖放在心上，把人民的利益放在首位。这些年来，围绕教育发展、社会保障、城市建设、农民增收以及公正司法、文明执法等关乎人民群众切身利益的热点、难点问题，常委会综合运用听取审议市政府专项工作报告、执法检查、办理代表议案和建议等多种手段，连续几年进行跟踪监督检查落实，促使“一府两院”努力在实现好、维护好和发展好人民群众的根本利益上下功夫，使发展成果让人民共享。

（四）必须坚持开拓进取，把创新意识贯穿人大工作始终。依法履行职责是人大工作的根本，解放思想、创新机制是做好人大工作的动力和取得实效的源泉。四届人大以来，创建了常委会专项报告“二审”制，促进了常委会审议质量的提高和审议意见的落实；推行了人代会期间专题审议制，提高了人代会审议质量；建立了信访联席会议制度，促进了一批“老大难”涉法信访案件的解决；开创了人大与纪委联合监督机制，使人大监督与党纪监督有机结合，增强了实效；实施了承诺公示和公示监督制度，增强和促进了领导干部的责任意识和勤政廉政建设；实行了被任命人员集体宣誓制度以及代表交叉视察等制度，增强了人事任免的严肃性，激励了代表视察的积极性。这些工作方式的创新和实践，使我市人大工作在依法行使职权的前提下，提高到了一个新的水平。

（五）必须坚持突出代表的主体地位，把代表工作贯穿人大工作始终。代表是国家权力机关的组成人员。几年来，常委会充分尊重代表的主体地位，自觉接受代表监督，注重调动代表执行职务的积极性，注重发挥代表联系人民群众的桥梁纽带作用，注重发挥代表在经济建设、政治建设、文化建设和社会建设中的骨干带头作用，为广大代表在人民代表大会期间认真审议报告、积极建言献策，闭会期间积极参加专题调研、视察和执法检查等活动创造条件、提供便利，使代表作用得以充分发挥，人大及其常委会的工作充满生机与活力。

（六）必须提高素质、强化服务，把加强自身建设贯穿人大工作始终。几年来，常委会机关坚持内强素质，外塑形象，自我加压，自我完善，相继提出了建设学习型机关、创新型机关、服务型机关、和谐型机关的目标，通过坚持常委会法制讲座和机关学习制度，有效提高了常委会组成人员及机关干部职工的综合素质；坚持制度建设不断线，使常委会机关在规范管理、改进服务上不断提升水平；逐步树立了风清气正、作风民主、务实高效的机关形象，保证了常委会各项工作的顺利开展和良好社会声誉。

今后一年的工作任务

2009年是新中国成立60周年，也是地方人大设立常委会30周年。做好今年的工作，对于积极应对挑战，推动我市各项事业实现新发展，充分发挥人民代表大会制度的优势和功效具有十分重要的意义。市人大常委会工作的指导思想和总体要求是：在中共晋城市委的领导下，坚持以邓小平理论和“三个代表”重要思想为指导，深入学习实践科学发展观，坚持党的领导、人民当家作主和依法治国的有机统一，紧紧围绕市委和政府的工作大局行使职权，突出重点，注重实效，切实发挥地方国家权力机关作用，为实现保增长、保民生、保和谐的目标作出新的更大的贡献。

一、以促进科学发展为中心，依法加强监督工作

要紧紧围绕市委工作部署，认真贯彻实施监督法，坚持以解决影响科学发展与社会和谐的突出矛盾和问题为重点，综合运用多种监督形式，督促和支持“一府两院”按照工作报告提出的目标任务做好各项工作，确保全年经济社会发展任务的顺利完成。

（一）加强专项工作审议监督。重点围绕转变经济发展方式、改善民生、推进法治、和谐建设，听取和审议市政府关于市五届人大五次会议代表建议办理情况的报告、关于生态园林城市建设情况的报告、关于公安工作情况的报告、关于全市村民委员会自治工作情况的报告、关于畜牧业发展情况的报告、关于推进科技进步情况等专项工作报告。通过审议监督，促进以上专项工作做好做实。

（二）加强计划、预算审议监督。听取和审议2008年市本级财政决算报告，批准2008年市本级决算；听取和审议2008年市本级财政预算执行情况和其他财政收支情况的审计报告及2009年上半年经济运行报告，保障计划预算按照人代会批准的目标任务健康运行。

（三）组织法律法规实施情况检查。重点围绕贯彻落实党的十七届三中全会精神和中共中央、国务院关于2009年促进农业稳定发展农民持续增收的若干意见，对《中华人民共和国土地管理法》的实施情况进行检查监督。重点检查我市合理利用、保护和开发土地资源情况，土地承包经营和土地流转情况，基本农田保护和违法案件查处情况，总结经验，查找问题，提出建议，督促法律实施主管机关加强和改进执法工作。同时，要配合组织好省人大常委在我市开展的各项执法检查。

（四）强化规范性文件备案审查。按照《晋城市人大常委会规范性文件备案审查暂行办法》，加强对市政府、县（市、区）人大常委会作出的规范性文件的备案审查工作，维护国家法制统一。

二、以发挥代表作用为主题，促进代表工作上台阶

深入贯彻中共中央〔2005〕9号文件精神，认真落实市人大常委会《关于加强和改进代表工作的意见》，积极探索代表工作的新路子，夯实代表工作基础，保障代表依法履行职责，充分发挥代表的主体作用。

（一）强化代表履职培训。组织代表举办新农村建设专题培训，指导县、乡人大搞好辖区内代表的履职培训工作，着力提升代表履职能力。推行代表履职登记制度，进一步完善代表履职的激励和监督机制。

（二）加强代表建议督办工作。坚持把办理代表建议与推进“一府两院”工作有效结合，发挥代表在代表建议督办工作中的作用。坚持和完善重点代表建议的领导领办、重点督办、跟踪督办制度，坚持办好《代表之声》栏目，运用群众、代表、新闻媒体等多种监督方式，努力提高代表建议办理工作水平。

（三）规范和丰富闭会期间的代表活动。加强对代表小组活动的指导和服务，坚持邀请代表列席常委会会议、参与常委会重要活动。组织各县（市、区）、市直单位代表交叉和集中视察，组织以关注边远山区和贫困农村为重点的专题视察活动，引导代表就关系全市经济社会长远发展和社会民生的热点、难点问题开展专题调研，提出议案和建议，发挥代表在推动全市经济平稳健康发展、保持社会和谐稳定中的重要作用。

（四）依法任免国家机关工作人员。坚持党管干部的原则，把充分体现市委意图和依法任免有机结合起来，坚持任前法律知识考试、供职表态、就职宣誓等制度，进一步规范工作程序，认真做好人事任免工作。坚持人大及其常委会选举任命干部的承诺公示、监督和年度述职制度，进一步提升任期监督的水平和实效。

三、以提高履职水平为目标，切实搞好调研宣传工作

调查研究是科学履职的基本功。提高履职水平必须深入开展调查研究，加强人大制度理论研讨和宣传工作，发挥理论研究和宣传工作对人大工作的指导和促进作用。

（一）坚持重点课题调研制度。全年共安排《律师法》实施情况、全市循环农业发展、全市农村文化建设、特色城镇化建设、代表小组活动等重点调研课题6项，努力以深入实际的调查研究，切实反映民意，科学集中民智，提高监督的实效性和履职水平。

（二）加强人大宣传工作。做好人代会、常委会及其重要活动的宣传服务工作，支持和协助新闻媒体加强和改进对人大工作的报道，运用各种新闻媒介，增强宣传的针对性和实效性，扩大人民代表大会制度的辐射面和影响力。继续办好《晋城人大》、太行日报《人大专刊》、晋城电视台《代表之声》专栏和《晋城人大网站》，提高人大工作透明度。今年是地方人大常委会设立30周年。我们要以纪念地方人大设立常委会30年为契机，认真总结和宣传我市人民代表大会制度建设和人大工作的经验，推动人大工作的理论创新和工作创新，使人民代表大会制度建设更加深入人心。

（三）开展法制专题宣传系列活动。今年要重点对新修订的《山西省丹河流域水污染条例》进行专题宣传，由常委会相关工作机构牵头组织开展一系列形式多样的宣传活动，拓宽广度，延伸深度，务求实效，让丹河条例真正成为我市治理丹河流域水污染的法制武器，推动全市水资源保护工作。

四、以提高干部队伍的素质为核心，不断加强自身建设

要坚持党的领导、人民当家作主和依法治国的有机统一，切实加强常委会及其机关的思想、作风和履职能力建设，不断强化干部队伍建设，不断提升人大工作质量和水平。

（一）进一步加强思想作风建设。以“服务科学发展，全面提高常委会机关集体参谋助手和服务保障工作水平”为载体，深入开展学习实践科学发展观主题活动，突出实践特色，深入总结经验，切实查找不足，认真进行整改，务求取得实效。

（二）进一步加强履职能力建设。坚持常委会学法和履职考核制度，组织开展多种形式的业务培训，增强履职意识，改进履职方式，提高履职能力和水平。

（三）进一步加强人大信访工作。健全信访工作机制，认真做好受理人民群众来信来访来电和申诉控告工作。坚持信访联席会议制度，提高涉法信访案件办理效率和质量。

（四）进一步加强常委会机关建设。继续加强机关制度建设、组织建设和干部队伍建设，巩固和谐机关创建成果，扎实推进文明机关创建活动，全面提升机关工作人员服务“三会”、服务代表的工作水平。

晋城的明天无限美好，人大的工作任重道远。让我们紧密团结在以胡锦涛同志为总书记的党中央周围，高举邓小平理论和“三个代表”重要思想伟大旗帜，全面贯彻落实科学发展观，在中共晋城市委的领导下，同心同德，开拓创新，为完成大会确定的各项目标任务而勤奋工作，为推动全市经济社会又好又快发展而努力奋斗，以优异的工作成绩迎接新中国成立60周年！
